# Biallelic *ELOVL1* Variants Are Linked to Hypomyelinating Leukodystrophy, Movement Disorder, and Ichthyosis

**DOI:** 10.1002/mds.30258

**Published:** 2025-07-01

**Authors:** Keit Men Wong, Reza Maroofian, Kolja Meier, Susann Diegmann, Tinatin Tkemaladze, Javeria Raza Alvi, Behnoosh Tasharrofi, Stephanie Efthymiou, Alexander Munchau, G. Christoph Korenke, Naif Almontashiri, Wafaa Eyaid, Amna Kashgari, Modhi Alotaibi, Jutta Gärtner, Brenda Huppke, Mostafa Asadollahi, Gocha Chikvinidze, Mohammad Keramatipour, Tipu Sultan, Holger Thiele, Peter Nürnberg, Markus H. Gräler, Henry Houlden, Peter Huppke

**Affiliations:** ^1^ Department of Neuropediatrics Jena University Hospital Jena Germany; ^2^ Center for Rare Diseases Jena University Hospital Jena Germany; ^3^ School of Pharmacy, University of Wyoming Laramie Wyoming USA; ^4^ Department of Neuromuscular Diseases Queen Square, Institute of Neurology, University College London London United Kingdom; ^5^ Department of Pediatrics and Pediatric Neurology and German Center for Child and Adolescent Health (DZKJ) University Medical Center Göttingen, Georg August University Göttingen Göttingen Germany; ^6^ Division of Clinical Genetics Givi Zhvania Pediatric University Clinic, Tbilisi State Medical University Tiblisi Georgia; ^7^ Department of Molecular and Medical Genetics Tbilisi State Medical University Tbilisi Georgia; ^8^ Department of Pediatric Neurology Children's Hospital and Institute of Child Health Lahore Pakistan; ^9^ Watson Genetic Laboratory Tehran Iran; ^10^ Institute of Systems Motor Science and Center for Rare Diseases University of Lübeck Lübeck Germany; ^11^ Section of Neonatology and Pediatric Intensive Care, Department of Pediatrics School VI‐School of Medicine and Health Sciences, Carl von Ossietzky Universität Oldenburg Oldenburg Germany; ^12^ College of Applied Medical Sciences and Center for Genetics and Inherited Diseases Taibah University Madinah Kingdom of Saudi Arabia; ^13^ Research Department King Khaled Eye Specialist Hospital Riyadh Saudi Arabia; ^14^ Department of Clinical Genetics and Precision Medicine King Abdulaziz Medical City Riyadh Kingdom of Saudi Arabia; ^15^ Department of Medical Genomics King Abdullah International Medical Research Center, King Saud Bin Abdulaziz University for Health Sciences Riyadh Kingdom of Saudi Arabia; ^16^ College of Medicine, King Saud Bin Abdulaziz University for Health Sciences Riyadh Kingdom of Saudi Arabia; ^17^ Department of Biology College of Science, Princess Nourah bint Abdulrahman University Riyadh Saudi Arabia; ^18^ Department of Child Neurology I. Tsitsishvili Children's New Clinic Tbilisi Georgia; ^19^ Cologne Center for Genomics (CCG) and Center for Molecular Medicine Cologne (CMMC), University of Cologne, Faculty of Medicine and University Hospital Cologne Cologne Germany; ^20^ Department of Anesthesiology and Intensive Care Medicine Jena University Hospital Jena Germany; ^21^ Center for Molecular Biomedicine (CMB), Jena University Hospital Jena Germany; ^22^ Center for Sepsis Control and Care (CSCC) Jena University Hospital Jena Germany

**Keywords:** ELOVL1, head tremor, ichthyosis, leukodystrophy, myoclonus

## Abstract

**Background:**

Very long chain fatty acids (VLCFAs) are an integral component of myelin and the epidermal water barrier. Variants in genes encoding enzymes responsible for catalyzing the first and rate limiting step in the production of VLCFAs, elongation of VLCFAs (ELOVLs), underlie a novel group of metabolic disorders.

**Objectives:**

The goal was to describe the clinical phenotype and disturbance in VLCFA metabolism associated with variants in the *ELOV1* gene.

**Methods:**

The following methods were employed: Exome sequencing, clinical phenotyping, magnetic resonance imaging (MRI), metabolomics, liquid chromatography–tandem mass spectrometry, fatty acid elongation assay.

**Results:**

We, here, describe seven patients with autosomal recessive variants in *ELOVL1*. Common clinical features included ichthyosis (5/7), developmental delay (7/7), progressive spasticity (7/7), nystagmus (5/6), and a complex movement disorder characterized by pronounced head tremor (7/7), myoclonus (6/7), and dysarthria (6/6). Brain MRI revealed non‐progressive hypomyelination (6/6) and hypoplasia of the corpus callosum (5/6). Plasma VLCFA analysis in one patient showed reduced concentrations of C24:0 and C26:0. Biochemical analysis of fibroblasts from this patient revealed elongation defects in VLCFA synthesis and dysregulation of other ELOVL enzymes.

**Conclusions:**

We show that biallelic variants in *ELOVL1* are associated with a unique and recognizable phenotype of hypomyelinating leukodystrophy, ichthyosis, and a complex movement disorder including progressive spasticity, head tremor, and myoclonus. Biochemical analyses confirmed a defect in VLCFA synthesis. Variants in genes encoding enzymes involved in the elongation of VLCFAs are a novel group of metabolic disorders with overlapping symptoms. © 2025 The Author(s). *Movement Disorders* published by Wiley Periodicals LLC on behalf of International Parkinson and Movement Disorder Society.

The coexistence of ichthyosis and neurological symptoms, referred to as neuro‐ichthyosis, can result from a range of rare genetic variants. Notably, some of these variants disrupt the synthesis of very long‐chain fatty acids (VLCFAs) (Rizzo et al^1^), which are crucial components of complex lipids involved in myelin formation and the maintenance of the epidermal water barrier. The synthesis of VLCFAs involves two primary pathways: one originating from dietary precursors and the other through de novo synthesis via fatty acid (FA) elongation. This elongation process takes place in the endoplasmic reticulum (ER) membrane through a four‐step cyclic process, including condensation, reduction, dehydration, and reduction, ultimately resulting in a fatty acid elongated by two carbons. The initial and rate‐limiting step is the condensation of acyl‐CoA with malonyl‐CoA, which donates two carbon units, leading to the production of 3‐ketoacyl‐CoA. This reaction is catalyzed by the elongase enzymes known as elongation of VLCFAs (ELOVLs). Currently, seven mammalian ELOVL proteins (ELOVL1‐ELOVL7) have been identified, each exhibiting characteristic yet partially overlapping substrate specificities toward acyl‐CoAs, as well as different tissue expression and developmental patterns.^2^, ^3^ ELOVL1 is active toward saturated and monounsaturated C20‐ to C24‐CoAs in vitro, therefore, contributing to the generation of C22 to C26‐VLCFAs.^4^ To date, only one pair of siblings with homozygous variants in *ELOVL1*, who presented with a pronounced congenital ichthyosis and a complex movement disorder, and two unrelated individuals with an identical heterozygous missense variant presenting with mild ichthyosis and progressive spasticity of the lower limb, have been reported.^5^, ^6^, ^7^ In this article, we describe the clinical findings in a further seven patients with homozygous variants in *ELOV1*, thereby expanding the known phenotypic spectrum and highlighting the variability of features associated with these variants. Furthermore, we demonstrate the biochemical consequences of one of the variants on VLCFA metabolism and summarize what is currently known about disorders associated with genes coding for ELOVLs.

## Patients and Methods

### Clinical Assessment

All cases were seen and examined by a pediatric neurologist and the videos were analyzed by an independent expert for movement disorders (A.M).

### Whole Exome Sequencing

DNA samples were collected from the index case and parents using EDTA blood after obtaining informed consent. The study was approved by the ethics commission at the University of Medical Center Göttingen (approval number 2516). Trio whole exome sequencing was conducted at the Cologne Center for Genomics (CCG, University of Cologne, Germany) (case 1) and at the UCL Institute of Neurology, London (cases 2–7), using methods described previously.^8^

### Cell Culture

Fibroblasts from case 1 were maintained as monolayer cultures in Dulbecco's modified Eagle's medium (Merck, Germany) (DMEM/low glucose) supplemented with 10% fetal bovine serum (FBS), 2 mM L‐glutamine and 100 U/mL penicillin, 100 μg/mL streptomycin. Cells were incubated at 37°C in an atmosphere of 5% CO2.

### Polymerase Chain Reaction and Sanger Sequencing

Polymerase chain reaction (PCR) of genomic DNA and complementary DNA (cDNA) sequences flanking c.462G>A variant of *ELOVL1* was performed using target‐specific primer sets. Detailed primer information is available in Supplementary Table S4. PCR products were purified and subsequently processed for direct dye terminator sequencing with BigDye Terminator Ready Reaction chemistry 3.1 on the Applied Biosystem 3500 genetic analyzer (Thermo Fisher, Germany).

### Quantitative Reverse Transcription Polymerase Chain Reaction

The quantitative reverse transcription polymerase chain reaction (RT‐qPCR) was performed as previously described.^8^ Data were normalized to *GAPDH*, used as a reference gene, and mRNA expression levels are presented relative to the control fibroblasts. Detailed primer information is available in Supplementary Table S4. All RT‐qPCR experiments were conducted with at least three independent replicates.

### Western Blotting

The western blotting was performed as previously described.^8^ Primary antibodies used in the study: C‐ELOVL1 (PA5‐63320, which detected peptide sequence 250–277, 1:1000 dilution), Anti‐FLAG M2 (Sigma, Germany, F1804; 1:1000 dilution), Calnexin (CS2679, 1:1000 dilution) and GAPDH (Abcam, United kingdom, 8245, 1:1000 dilution).

### Immunofluorescence

Fibroblasts or transfected HeLa cells were seeded and grown on coverslip. Next day, cells on coverslips were fixed in cold 4% paraformaldehyde for 20 minutes at room temperature (RT). Immunofluorescence on fibroblasts or HeLa cells was performed using standard procedures. Briefly, after post‐fixation, cells were washed three times with cold phosphate‐buffered saline and permeabilized with 0.05 M Tris‐buffered saline (TBS) solution containing 0.5 M ammonium chloride and 0.25% Triton X‐100 for 10 minutes. Cells were then washed with 0.05 M TBS solution, blocked with 5% normal horse serum in 0.05 M TBS for 1 hour at RT and incubated with indicated primary antibodies overnight at 4°C. After washing, secondary antibodies were applied for 1 hour and the coverslips with cells were mounted onto the SuperFrost plus slide (Thermo Fisher Scientific, Germany) with proLong gold antifade reagent with 4′,6‐diamidino‐2‐phenylindole (Invitrogen Ref P36935). The stained cells were analyzed using fluorescent microscopy with Apotome.2 (Zeiss, Germany).

### Metabolomics Analysis with the AbsoluteIDQ p‐180 Kit

Metabolomics analysis was performed using the AbsoluteIDQ p180‐Kit (Biocrates Lifesciences, Innsbruck) applied to a BEH Amide column (Waters, Milford, MA) and a Xevo TQ‐S mass spectrometer (MS) (Waters). The kit was processed using the guidelines of the manufacturer. Measurement of the kit consisted of two separate analyses, flow injection analysis (FIA‐MS) for lipids and liquid chromatography MS (LC–MS) for amines. FIA‐MS was used for the quantification of up to 145 metabolites (including 40 acylcarnitines, 15 sphingolipids, 76 phosphatidylcholines, and 14 lysophosphatidylcholines). LC–MS analysis includes the measurement of 21 canonical amino acids and 21 other biogenic amines.

Briefly, 10 μL patient sample was applied to the kit plate that had been lined with an internal standard solution and dried using the positive pressure manifold (Waters, Milford, United States). Consecutively, the dried sample plate was treated with phenylisothiocyanate to derivatize amines and dried under nitrogen flow. Samples from the plate were extracted using 5 mM ammonium acetate in methanol and diluted in flow injection analysis solvent for FIA analysis and in water for LC–MS analysis, respectively. FIA and LC–MS runs were performed. LC–MS data were first analyzed using MassLynx V4.1 software (Waters) using the supplied analysis method by Biocrates (Innsbruck, Austria), confirmed manually, and imported into the software supplied by the kit (MetIDQ Carbon, Biocrates). FIA data were directly loaded into MetIDQ and interpreted automatically.

### Analysis of Lipid Profiles by LC–Tandem MS

Cells were resuspended in 200 μL methanol for protein precipitation. After addition of an internal standard mix (C17‐sphingomyelin, C15‐ceramide, C12‐glucosylceramide, 30 pmol of each), samples were stored overnight at −80°C and centrifuged at 30,000 *g*. The supernatant was transferred into 1.5 mL autosampler vials equipped with 200 μL glass inserts. Quantifications were performed by LC–tandem MS (MS/MS). The Prominence high‐performance liquid chromatography (HPLC) system consisting of controller CBM‐20A, automatic sampler SIL‐20 AC, column oven CTO‐20A, pump LC‐20 AD, and degasser LC‐20A5R (Shimadzu, Duisburg, Germany) was used to separate the analytes. A total of 50 μL sample volume was injected onto a MultoHigh 100 RP18‐3 60 × 2 mm column (CS Chromatographie Service, Langerwehe, Germany) that was used at 35°C under the indicated conditions (Supplemental Table S5). Mass spectrometric detection was performed using QTrap triple quadrupole mass spectrometer in multiple reaction monitoring (MRM) mode and positive ionization at 450°C (AB Sciex, Darmstadt, Germany) using the specific m/z of original masses in quadrupole Q1 and the fragments m/z 184.1 for sphingomyelins and m/z 264.4 for ceramides and hexosylceramides in Q3. Samples were quantified using Analyst 1.6.3 (AB Sciex, Darmstadt, Germany) relative to the added amount of internal standards.

### Generation of ELOVL1 Plasmids

Construction of the 3xFLAG‐*ELOVL1‐*WT or 3xFLAG‐*ELOVL1‐*(p.W154*) plasmids were generated through OriGene Technologies (Rockville, MD, United States). In brief, for expressing of N‐terminally 3XFLAG‐tagged proteins, the full human *ELOVL1* (NM_022821) open reading frame (cat. RC200027) that contained restriction site *Sgfl* and *Mlul* were subcloned into pCMV6‐AN‐3DDK vector (cat. PS100058), therefore, producing 3XFLAG‐*ELOVL1*‐WT. The 3xFLAG‐*ELOVL1‐*(p.W154*) plasmid was created by site‐directed mutagenesis using PCR with mismatched primer 5′‐ TTCCCTGGAGCTGGTGGTG**A**GGGGTAAAGATTGCCCCGG‐3′ (introduced mutation c.462G>A in bold). All constructs were verified by sequencing.

### FA Elongation Assay

The assay was performed using the total membrane fraction as previously described with some modifications.^4^ HeLa cells were transfected with either with 3xFLAG‐*ELOVL1‐WT* or 3xFLAG‐*ELOVL1*(p.W154*) construct using Effectene (Qiagen), according to manufacturer's instructions. Cells were suspended in buffer A (50 mM Hepes‐NaOH (pH 7.5), 150 mM NaCl, 10% glycerol, 1 mM DTT, 2 mM MgCl2, 1 mM CaCl2, 1x protease inhibitor mixture (Complete, EDTA‐free, Roche Diagnostics, Germany), and 1 mM PMSF and lysed by sonication. After centrifugation at 100,000 × *g* at 4°C for 30 minutes, the pellet (total membrane fraction) was resuspended in buffer A and subjected to the fatty acid elongation assay. Total membrane fractions (10 μg of protein) were incubated with 100 μM [13C] malonyl‐CoA (Sigma) and 10 μM of C22:0‐CoA (behenoyl‐CoA) (Avanti Polar Lipids, Alabaster, AL, United States) complexed with 0.2 mg/mL fatty acid‐free bovine serum albumin in buffer A containing 1 mM NADPH at 37°C for 30 minutes. The reactions were terminated by the addition of 10 volume of methanol. Lipids were extracted and analyzed by LC–MS/MS as described below.

### Analysis of Fatty Acid Elongation by LC–MS/MS

Ten volumes of methanol were added to the reaction mixtures for protein precipitation. After addition of 10 pmol arachidonic acid‐d8 as internal standard (Cayman, Ann Arbor, MI, United States), samples were stored overnight at −80°C and centrifuged at 30,000 *g*. The supernatant was transferred into a glass centrifuge tube and evaporated using a vacuum concentrator (Christ, Osterode, Germany). Samples were resuspended in 50 μL methanol and transferred into 1.5 mL autosampler vials equipped with 200 μL glass inserts. Quantifications were performed by HPLC coupled with triple quadrupole mass spectrometry (LC–MS/MS). The Agilent series 1200 HPLC system was used to separate the analytes (Agilent, Santa Clara, CA, United States). A total of 30 μL sample volume was injected onto a Hamilton PRP‐1 5 μm 100 × 2.1 mm column (CS Chromatographie Service, Langerwehe, Germany), which was used at 35°C at a flow rate of 200 μL with 10% of eluent A (methanol), 10% eluent B (5% ammonium water), and 80% eluent C (water), switching to 90% A and 10% B after sample injection. Eluent mixture switched back to 10% A, 10% B, and 80% C after 13 minutes. The program ended after 20 minutes. Mass spectrometric detection was performed using QTrap triple quadrupole mass spectrometer in MRM mode using pseudofragmentation and negative ionization at 450°C (AB Sciex, Darmstadt, Germany). The detected mass transitions were m/z 311.3/311.3 (arachidonic acid‐d8) and m/z 369.3/369.3 ([^13^C]_2_‐tetracosanoic acid). Analyses were performed with Analyst 1.6.3 (AB Sciex) relative to the added amount of internal standard.

### Statistical Analysis

Data of metabolites (AbsoluteIDQ p‐180 kit) were exported from MetIDQ and metabolites above the lower limit of quantification were subjected to statistical analysis using MetaboAnalyst 5.0. Data were normalized using log transformation and auto‐scaling. Statistical analysis of the mRNA expression or sphingolipids concentration in fibroblasts was performed using the Prism 10 (GraphPad software). Statistical differences were considered to be significant when *P* < 0.05. All quantitative data were presented as mean (±standard deviation [SD]).

## Results

### Clinical Review of *ELOVL1* Cases

A summary of the clinical findings, including those from the four previously described cases, is presented in Supplementary Table S1. Videos of cases 1 and 4 are shown in the supplement ([Link], [Link], [Link], [Link]). Case reports of cases 1 to 5 are included in the Supplementary Case Reports. Seven cases from five unrelated families were included in the study, four of whom were female. The age at last presentation ranged from 4 to 21 years. All cases originated from healthy consanguineous parents). Pregnancy and birth were unremarkable in all. Congenital ichthyosis was present in five cases, affecting the limbs and the trunk (Fig. 1A). Motor development was primarily delayed in all cases, although all achieved unsupported sitting by 16 months of age. Three cases achieved unsupported walking. Progressive spasticity, predominantly in the lower limbs, occurred in all cases, leading to loss of walking in one. Additionally, all cases exhibited a movement disorder characterized by axial hypotonia (6/6), head tremor (7/7), dystonia (4/7), myoclonus (6/7), and dysarthria (6/6). Myoclonic jerks were observed at rest and became more pronounced with voluntary movement. In some patients, these jerks significantly impaired ambulation. Although a tremor involving the head was most prominent, in several cases the upper limbs were also affected. Mild intellectual disability was present in four of five cases, however, communication skills were preserved in all. Two cases had photophobia, and five of six exhibited nystagmus. Hearing impairment, as described in all four previously reported cases, was not present.

**FIG. 1A:**
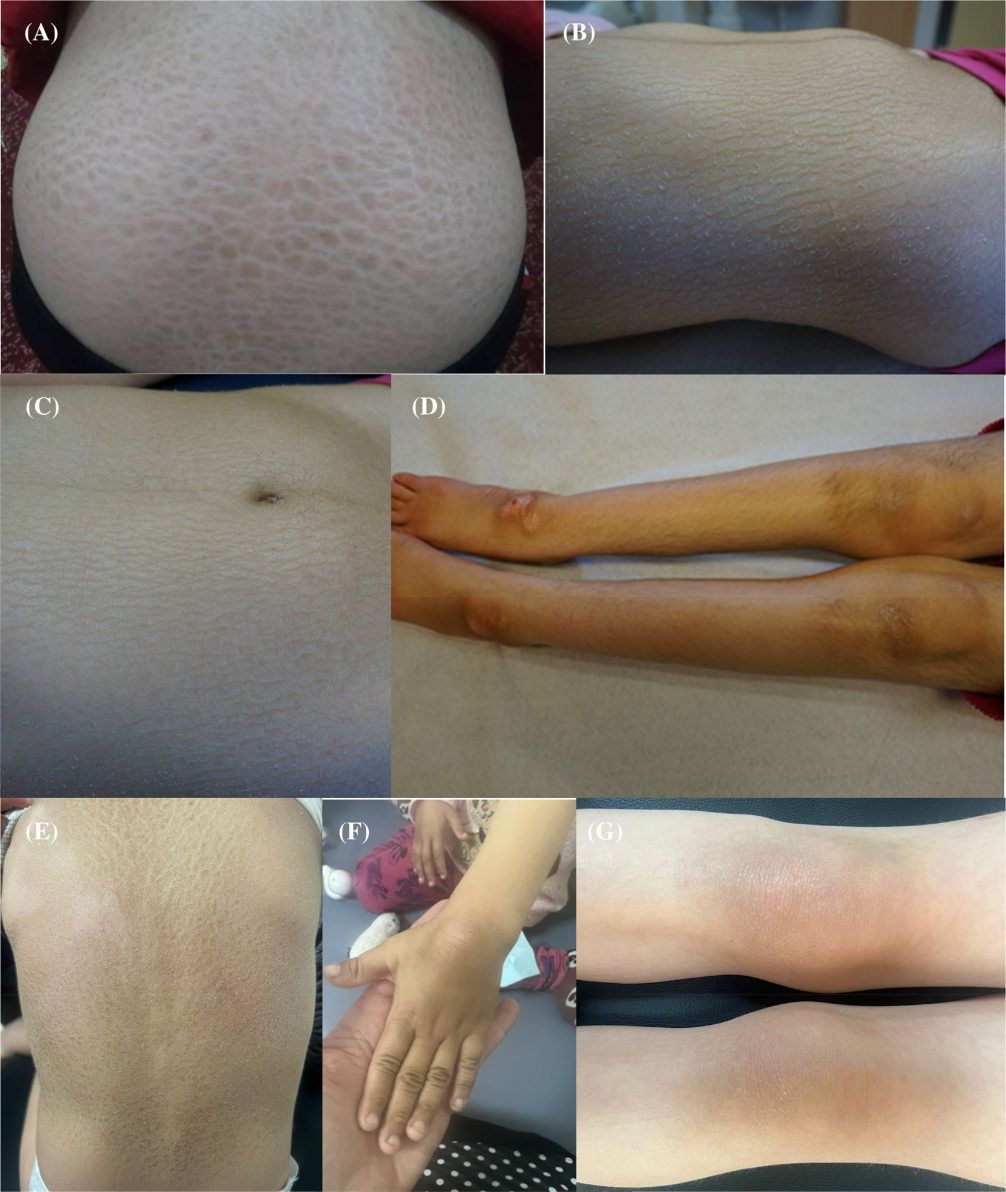
Clinical phenotypes and magnetic resonance (MR) images of the ELOVL1 patients. (A) Images from the back of case 5; (B,C,D) the trunk, abdomen and legs of case 1; (E,F,G) and the back, hand and legs of case 4.

**FIG. 1B:**
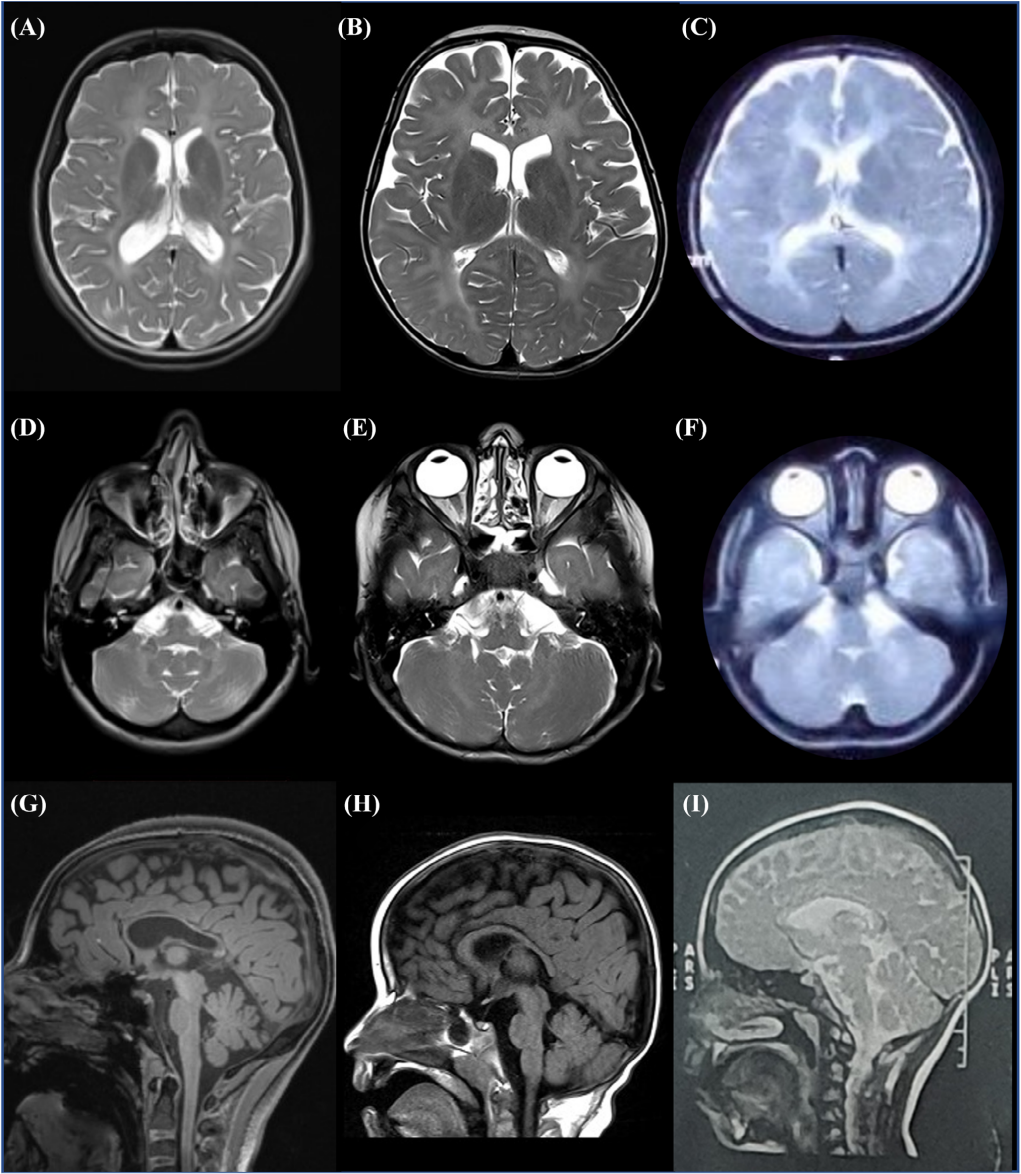
T2 weighted MR images from cases 1, 2, 4, and 5 demonstrating hypomyelination of the cerebrum (**A–C**) and cerebellum (**C–F**) and T1 weighted images showing hypoplasia of the corpus callosum (**G–I**). (**A,D,G**) Case 1 at age 14; (**B,E,H**) case 4 at age 5 years; (**C,F**) case 2 at age 6 years; and (**I**) case 5 at age 13 years.

Magnetic resonance imaging (MRI) of the brain revealed hypomyelinating leukodystrophy (6/6), also affecting the cerebellum and hypoplasia of the corpus callosum (5/6) (Fig. 1B). Hypomyelination was independent of age at imaging and repeated magnetic resonance imaging in case 1 showed no progression.

Analysis of VLCFAs in plasma was performed only in case 1 and demonstrated a decrease in C24:0 and C26:0 concentrations, consistent with the function of ELOVL1 in the synthesis of C22‐C26 VLCFAs (Supplementary Table S2).

### Molecular Genetic Data of *ELOVL1* Variants

**FIG. 2:**
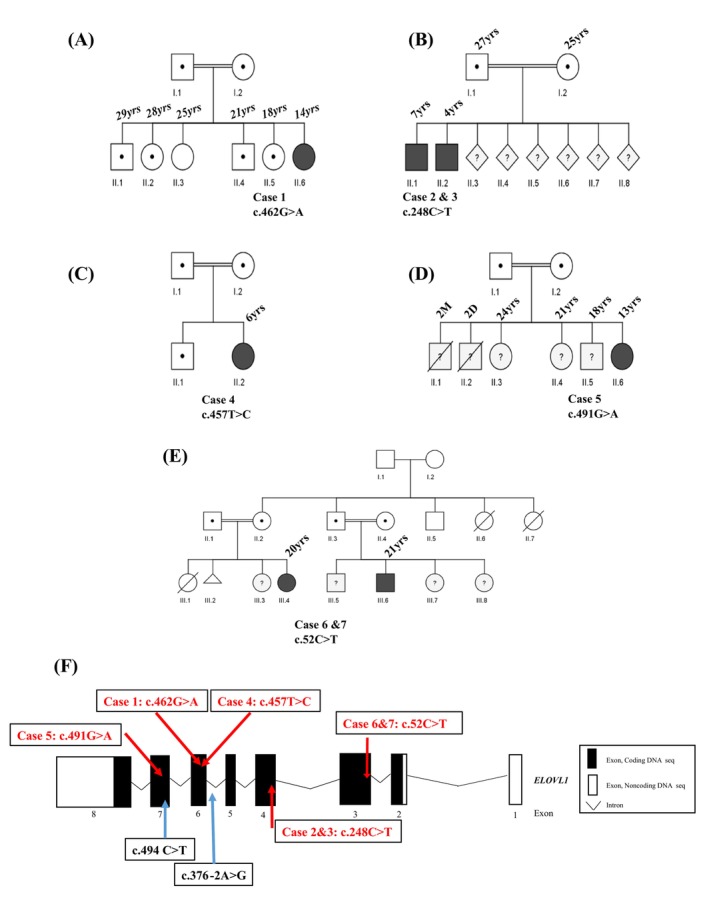
Pedigree and gene structure of the families with ELOVL1 variants. (**A**) Pedigree of the Case 1 family. (**B**) Pedigree of the Case 2 and 3 family. (**C**) Pedigree of the Case 4 family. (**D**) Pedigree of the Case 5 family. (**E**) Pedigree of the Case 6 and 7 family. (**F**) Gene structure of ELOVL1. The positions of the variants reported in this study are indicated by red arrows, and previously published variants are indicated by blue arrows. [Color figure can be viewed at wileyonlinelibrary.com]

The cases included in this study were assembled via a collaboration using Genematcher.^9^ Exome sequencing was done as part of a routine clinical genetic analysis and revealed homozygous variants in *ELOVL1* (NM_022821.3): c.462G>A (p.Trp154*) in case 1; c.248C>T (p.Ser83Leu) in cases 2 and 3; c.457 T>C (p.Trp153Arg) in case 4; c.491G>A (p.Gly164Asp) in case 5; and c.52C>T (p.Arg18Trp) in cases 6 and 7 (Fig. 2). The frequency of the reported ELOVL1 variants in the general population is extremely low according to gnomAD v4.1.0 (see Supplementary Table S3). Variants identified in cases 1 to 4 were confirmed by Sanger sequencing (Supplementary Fig. S1). Sanger sequencing of the *ELOVL1* gene in the immediate family of case 1 confirmed segregation with the disorder. All variants detected in *ELOVL1* are predicted to be pathogenic by different conservation and functional impact scores and are located in highly conserved amino acid residues (Supplementary Fig. S2 and Supplementary Table S3). According to American College of Medical Genetics and Genomics (ACMG) guidelines the ELOVL1 variant c.462G>A would be classified as pathogenic (PVS1_VS, PM2_P, PM3_P), the c.248C>T variant as a variant of uncertain significance (VUS) (PM1_P, PM2_P, PP3_0, PM3_M), the c.457 T>C variant as a VUS (PM1_P, PM2_P, PM3_P, PM3_P), the c.491G>A variant as a VUS (PM1‐P, PM2_P, PM3_P, PM3_0), and the c.52C>T variant also as a VUS (PM1_P, PM2_P, PM3_M, PP3_0).^10^ Exome sequencing included all other genes associated with hypomyelinating leukodystrophy. No pathogenic variants were detected in these genes.

### Expression of *ELOVL1* p.Trp154X Variant in Case 1 Fibroblasts

**FIG. 3:**
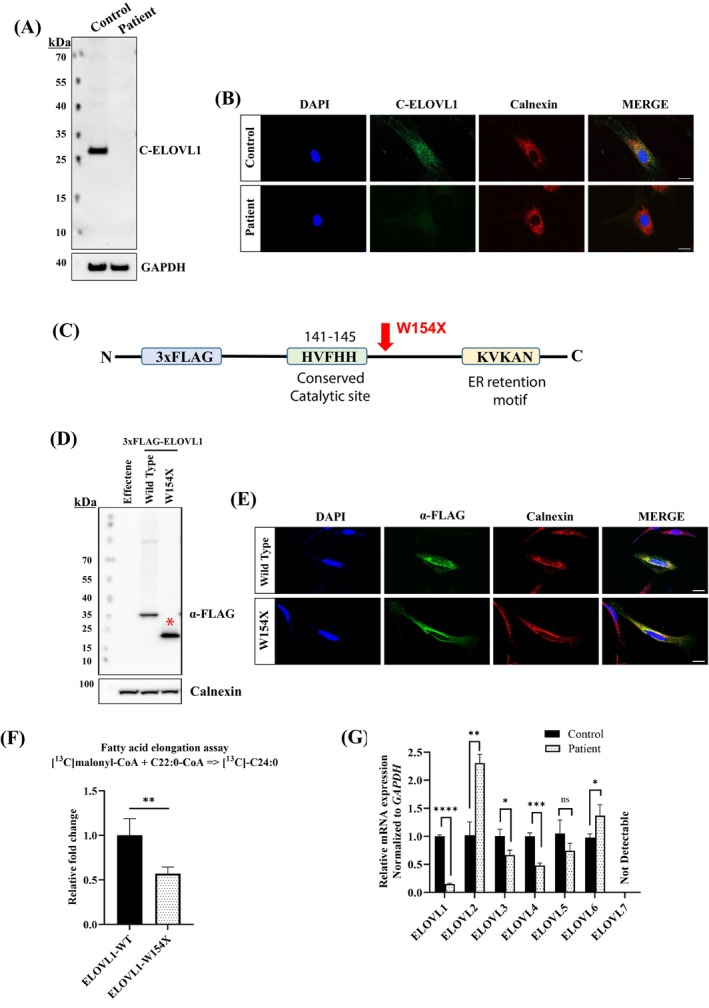
Biochemistry analysis of the *ELOVL1* Case 1 carrying c.462G>A variant. (**A**) Western blot analysis of control and patient fibroblasts probed with the indicated antibodies for C terminal of ELOVL1 (C‐ELOVL1) and GAPDH as loading control. Note no truncated proteins were detected. (**B**) Representative Immunofluorescence for C‐ELOVL1 (green) and endoplasmic reticulum (ER) marker, Calnexin (red) in control and patient 1 fibroblast. C‐ELOVL1 was colocalized with Calnexin in control fibroblast while it was not detectable in the patient's fibroblast. Scale bars, 20 μM. (**C**) Human ELOVL1 construct illustrating FLAG‐tagged ELOVL1‐wild type (WT) and the ELOVL1‐W154X mutant. (**D**) Western Blot analysis of cell lysates from transfected HeLa cells expressing ELOVL1‐WT and ELOVL1‐W154X mutant construct. Wild type ELOVL1 protein is expressed at the predicted size 37 kDa, whereas a truncated protein (asterisk) is observed at ~20 kDa in ELOVL1 mutant construct. (**E**) HeLa cells transfected with ELOVL1‐WT and W154X mutant were immunostained for FLAG (green) and ER marker Calnexin (red). ELOVL1 mutant showed similar colocalization to WT with ER marker. Cells were counterstained with 4′,6‐diamidino‐2‐phenylindole (DAPI) (blue) to show nuclei. Scale bars, 20 μM. (**F**) Relative ELOVL1 enzymatic activity in transfected HeLa cells expressing ELOVL1‐WT and ELOVL1‐W154X construct. ELOVL1‐W154X mutant shows reduced elongation activity of C22:0‐CoA when compared to WT. Data are given as means ± standard deviation (SD), n = 3. Statistical differences were obtained with unpaired student's *t* test: *P ≤ 0.05, **P ≤ 0.01. (**G**) Quantitative reverse transcription polymerase chain reaction (RT‐qPCR) analysis of *ELOVL1* and other *ELOVL* isozymes mRNA expression in primary fibroblast cell line from control and patient 1 with *ELOVL1* c.462G>A variant. Expression is normalized to that of *GAPDH*. Percentage of mRNA is equal to 2 − ΔΔCT and normalized relative to control. Data are given as means ± SD, n ≥ 3 independent experiments. Statistical differences were obtained with unpaired Welch's *t* test: *P ≤ 0.05; **P ≤ 0.002; ***P ≤ 0.0002; ****P ≤ 0.0001, ns, not significant. Note ELOVL7 is not detectable in skin fibroblast. [Color figure can be viewed at wileyonlinelibrary.com]

As expected for a nonsense variant, Western blot analysis using a polyclonal antibody targeting the C‐terminal peptide sequence of ELOVL1 (amino acids 250–277, C‐ELOVL1) detected no signal in whole cell extracts from case 1 fibroblasts (Fig. 3A). Consistent with these findings, immunofluorescent micrographs demonstrated the expected colocalization of C‐ELOVL1 with the endoplasmic reticulum marker Calnexin in control fibroblasts. In contrast, no such immunoreactivity was observed in the fibroblasts of case 1 (Fig. 3B).

### Subcellular Localization and Enzymatic Activity of the p.Trp154X *ELOVL1* Variant

To investigate the subcellular localization and elongation activity of the *ELOVL1* variant detected in case 1, we generated two constructs: one representing the wild type (ELOVL1‐WT) and the other representing the variant (*ELOVL1*‐W154X). These constructs were fused with a triple N‐terminal FLAG tag (Fig. 3C). Western blot analysis of transfected HeLa cells revealed the presence of a truncated ELOVL1 protein in *ELOVL1*‐W154X cells (Fig. 3D). Unexpectedly, despite the lack of the ER retention signal, both the WT and mutant constructs exhibited perinuclear staining, co‐localizing with the ER marker Calnexin (Fig. 3E). However, an in vitro elongation assay conducted on *ELOVL1*‐W154X demonstrated a substantial reduction in enzymatic activity compared to *ELOVL1*‐WT (Fig. 3F).

### Expression of ELOVL1 and Other ELOVLs in Fibroblasts from Case 1

Quantitative real‐time PCR revealed a substantial reduction in ELOVL1 mRNA in case 1, possibly because of nonsense‐mediated decay (Fig. 3G). Analysis of mRNA levels for other known ELOVL enzymes showed a significant decrease in ELOVL3 and ELOVL4 expression, along with a notable increase in ELOVL2 and ELOVL6 mRNA expression, indicating widespread dysregulation of VLCFA metabolism because of the variant (Fig. 3G). ELOVL7 was undetectable in skin fibroblasts of both case 1 and controls, consistent with previous reports.^11^, ^12^

### Sphingolipids Alteration in Plasma and Fibroblasts of Case 1

**FIG. 4:**
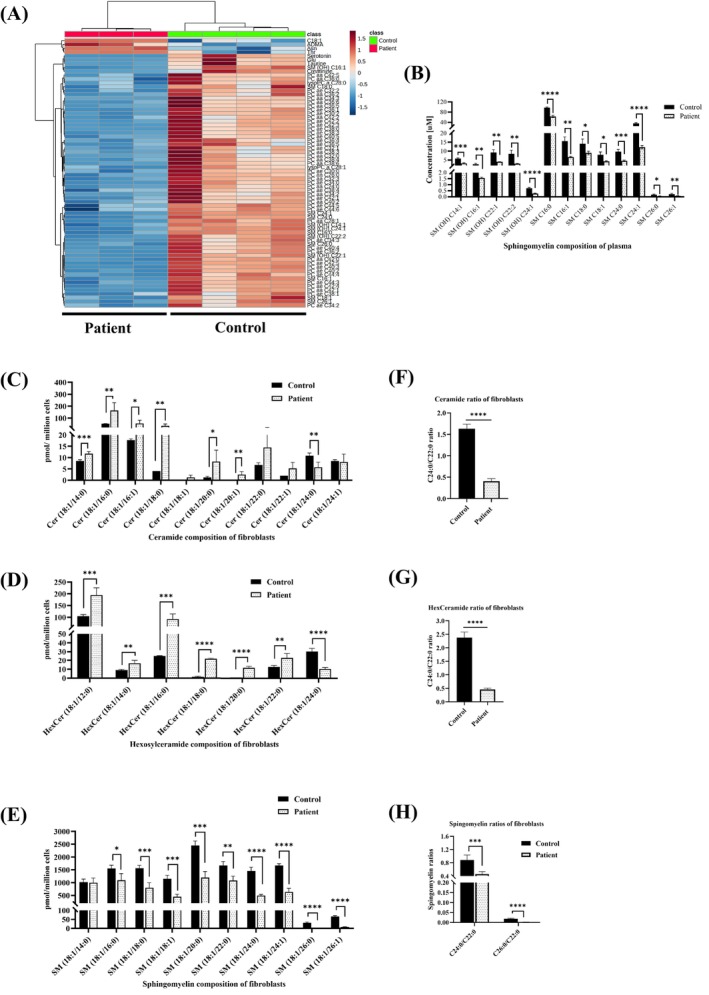
Metabolic alterations of the *ELOVL1* Case 1 carrying c.462G>A variant. (**A**) Heatmap clustering of the control and patient's plasma. Plasma from patients analyzed using the Biocrates AbsoluteIDQ p180 kit. Euclidean distance and Ward clustering were used. (**B**) Concentration of various Sphingomyelin composition of the control and patient's plasma. (**C**) Ceramide composition of control and patient's fibroblasts. (**D**) Hexosylceramide composition of control and patient's fibroblasts. (**E**) Sphingomyelins composition of control and patient's fibroblasts. (**F**) Ceramide ratio, (**G**) hexosylceramide ratio and (**H**) sphingomyelins ratio of fibroblasts. Data are given as means ± standard deviation. Statistical differences were obtained with unpaired student's *t* test: *P ≤ 0.05; **P ≤ 0.01; ***P ≤ 0.001; ****P ≤ 0.0001. [Color figure can be viewed at wileyonlinelibrary.com]

A comprehensive metabolomics analysis using the AbsoluteIDQ p‐180 kit and heatmap clustering revealed significant perturbations in the metabolism of complex lipids, particularly sphingolipids and glycerophospholipids, within the patient's plasma (Fig. 4A). Specifically, all sphingomyelins (SMs) exhibited a substantial decrease in the plasma of case 1 (Fig. 4B).

To analyze the effect on the acyl chain lengths of sphingolipids, we evaluated the levels of ceramides (Cer), hexosylceramides (HexCer), and sphingomyelins (SM) in fibroblasts derived from case 1. We found reduced levels of VLCFA C24:0‐ceramides and C24:0‐HexCer, coupled with an increase in shorter chain lengths of Cer and HexCer (Fig. 4C,D). Consistent with the plasma results, the majority of SMs, excluding C14:0‐SM, displayed a substantial reduction, underscoring the impact of the ELOVL1 defect (Fig. 4E). This was further evidenced by a decline in the C24:0/C22:0 ratios of Cer and HexCer, as well as the C24:0/C22:0 and C26:0/C22:0 ratios of SM (Fig. 4F–H). Overall, these results strongly suggest that the ELOVL1 c.462G>A variant leads to a shortening of the acyl chain lengths of VLC‐sphingolipids.

## Discussion

Genetic defects affecting the production and elongation of VLCFAs are a novel group of metabolic disorders. In this article, we describe the phenotype and biochemical abnormalities associated with homozygous *ELOVL1* variants. All variants described in this article have not been described previously and are ultra‐rare in the general population and affect highly conserved amino acid residues throughout vertebrates. In silico prediction programs suggest that these variants are likely pathogenic. According to the ACMG guidelines, only the variant identified in case 1—a homozygous stop‐gain variant—meets the criteria to be classified as likely pathogenic. The remaining missense variants are currently considered VUS, although they are highly suggestive of pathogenicity because of their segregation within affected families and the strong phenotypic specificity observed. These findings support the possibility that the missense variants are causative. However, further biochemical validation and identification of additional patients with similar variants will be essential to confirm their pathogenic role.

**FIG. 5:**
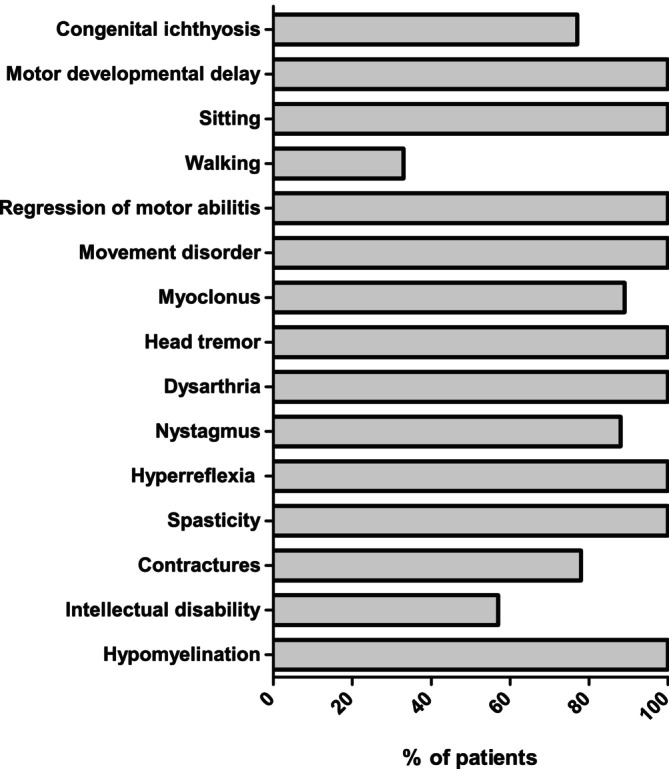
Summary of the clinical features seen in the patients with biallelic variants in ELOVL1.

Despite a similar neurological phenotype in all 11 cases described to date with either homozygous (n = 9) or heterozygous (n = 2) variants in *ELOVL1*, the ichthyosis phenotype is highly variable (clinical features are summarized in Fig. 5). Cases 2 and 3 with homozygous variants did not exhibit ichthyosis, whereas the others showed involvement of the trunk and limbs. For those with ichthyosis, it was present at birth in the cases with homozygous *ELOVL1* variants while it developed later during the first year of life in the two cases with heterozygous variants.

All cases showed delayed motor development and axial hypotonia, but all achieved unsupported sitting and three were able to walk at least temporarily. Common neurological findings that were present from early childhood were nystagmus and dysarthria. In all cases, slowly progressive spasticity was described leading to contractures. A pronounced movement disorder with prominent head tremor, dystonia, and myoclonus was notably only present in the nine cases with homozygous variants. Despite severe neurological symptoms, intellectual disability was only present in four cases, all with homozygous variants, and described as mild and non‐progressive.

MRI findings were consistent across all cases, revealing pronounced non‐progressive hypomyelinating leukodystrophy. Hypomyelinating leukodystrophies are a group of disorders resulting from primary defects in myelin caused by genetic defects in structural proteins, transcription factors, lysosomal enzymes, and RNA translation. The clinical presentation of the cases with ELOV1 variants closely aligns with typical features of hypomyelinating leukodystrophies, such as Pelizaeus‐Merzbacher disease (PMD), characterized by congenital nystagmus, motor impairment, ataxia, and spasticity, with comparatively milder cognitive impairment.^13^ Similar to PMD, patients harboring homozygous *ELOVL1* variants exhibit a slowly progressive clinical course marked by worsening dysarthria and spasticity. However, the movement disorder, including head tremor and myoclonus present in *ELOVL1* patients would be atypical for a hypomyelinating leukodystrophy like PMD. *TUBB4A*‐related disorder is an exception as it often manifests with severe dystonia. However, it is frequently accompanied by basal ganglia and cerebellar atrophy (hypomyelination with atrophy of the basal ganglia and the cerebellum [H‐ABC]), which was not observed in the patients described here.^14^ Several hypomyelinating leukodystrophies present with involvement of organs outside the central nervous system (CNS) that can be diagnostic. For example, abnormal dentition is seen in 4H leukodystrophy and occulodentodigital dysplasia, whereas bone involvement is seen in hypomyelination with spondylometaphyseal dysplasia.^13^ To the best of the authors' knowledge, the co‐occurrence of ichthyosis and hypomyelination has previously only been described in *ELOVL4*‐related disorders; however, these cases did not exhibit movement disorders. The triad of hypomyelinating leukodystrophy, ichthyosis, and a movement disorder appears to be unique to *ELOVL1*‐related disorders. Within the cohort, we observed a range of phenotypic variability, even among individuals carrying the same genetic variant. The sample size was too small to establish a definitive genotype–phenotype correlation. However, the two identified nonsense variants did not appear to be associated with a more severe clinical presentation. This variability suggests that genetic modifiers may contribute significantly, given the complex nature of VLCFA metabolism. Additionally, environmental factors—particularly dietary VLCFA intake—may also influence disease expression. Future studies should address these aspects, as they may have important therapeutic implications.

In an attempt to better understand the pathophysiology of this novel disorder, we analyzed plasma and skin fibroblasts from case 1. Unfortunately, fibroblasts were unavailable from the other cases. The *ELOVL1* gene encodes a protein of 279 amino acids with seven transmembrane‐spanning domains, including a histidine cluster motif (HXXHH) crucial for enzymatic activity, and an ER retention signal composed of a di‐lysine motif (KXKXX) located at the C‐terminus.^15^ The c.462G>A (p.Trp154*) variant introduces a premature stop codon, which is predicted to disrupt ELOVL1 function either by triggering nonsense‐mediated mRNA decay (NMD) or by producing a truncated protein. Our findings suggest that NMD is playing a role, at least to some extent, as evidenced by markedly reduced transcript levels (Fig. 3G). However, it is possible that both NMD and truncated protein production are contributing to the observed phenotype. In the event that a truncated protein (p.W154X) is expressed, it may retain the histidine cluster motif but exhibit abnormal localization. Interestingly, our biochemical analysis, using a FLAG antibody in transfected cells, revealed that the *ELOVL1*‐W154X variant localizes to the ER. The ER localization of the W154X variant, despite lacking the canonical ER retention signal, is intriguing. One possible explanation is that the truncated protein retains membrane‐spanning domains that may promote ER retention.^16^, ^17^ Cannon and Cresswell^18^ showed that incomplete assembly of transmembrane domains can cause ER retention, which may similarly apply to the truncated protein in our study. Additionally, although speculative, retention could occur through indirect mechanisms such as association with other ER‐localized components. It has also been reported that FLAG tags may influence protein localization,^19^ which cannot be excluded in our experimental setup. Future studies will be needed to address these possibilities, including the use of constructs with alternative tags as well as computational prediction tools to assess whether the remaining transmembrane domains of the truncated protein mediate ER anchoring.

Comprehensive analysis of lipid composition in the patient's plasma and skin fibroblasts highlighted a significant reduction in very long‐chain acyl constituents within sphingolipids, consistent with impaired ELOVL1 function. Notably, despite its ER localization, the *ELOVL1*‐W154X variant consistently displayed a deficiency in FA elongation, akin to what has been reported for the S165F variant of ELOVL1 protein.^6^ Based on these findings, we propose that the truncated protein may have an altered secondary structure or a reduced capacity to interact with components of the elongation machinery, thereby compromising VCLFA synthesis. However, this remains speculative, as no direct structural or interaction data were obtained. Further studies incorporating structural modeling, circular dichroism spectroscopy, or co‐immunoprecipitation will be essential to test this hypothesis and clarify the underlying mechanism.

The ELOVL enzyme family can be categorized into two primary groups. ELOVL1, ELOVL3, ELOVL6, and ELOVL7, primarily elongate saturated and monounsaturated VLCFAs, and ELOVL2 and ELOVL5 are primarily involved in the elongation of polyunsaturated fatty acids. ELOVL4 is an exception as it has been found to be active in both groups.^20^ Analyzing the expression of other ELOVL enzymes, Mueller et al^6^ found an upregulation of only *ELOVL2* mRNA in S165F skin fibroblasts. In skin fibroblasts of case 1 who carries the homozygous recessive W154X *ELOVL1* variant, we also observed an upregulation of *ELOVL2* but also *ELOVL6* mRNA, along with significant downregulation of *ELOVL3* and *ELOVL4* mRNA. These results indicate that the expression of ELOVL enzymes are interconnected, but one can only speculate if the changes in expression we found are signs of a compensatory upregulation or a dysregulation because of abnormal intermediates in the VLCFA metabolism.

Variants in two further ELOVL enzymes, ELOVL4 and ELOVL5, have been associated with disorders (Table 1). *ELOVL4*‐related ichthyosis, spastic quadriplegia, and mental retardation (MIM 614457) an autosomal recessive disorder, characterized by ichthyosis, global developmental delay, epilepsy, and failure to thrive, has so far been described in nine families.^21^, ^22^, ^23^, ^24^ Dominant variants in *ELOV4* on the other hand give rise to Stargardt disease 3 (MIM 600110),^25^ a macular dystrophy that typically manifests in the second decade of life, and spinocerebellar ataxia‐34 (MIM 133190), characterized by slowly progressive ataxia, dysarthria, nystagmus, and in some cases, hyperkeratosis.^26^ Heterozygous *ELOVL5* variants have been associated with spinocerebellar ataxia 38 a disorder characterized by adult onset progressive ataxia, dysarthria, and nystagmus. It has been described so far in three Italian and one French family (Di Grgoiro). From these disorders autosomal recessive *ELOVL4* variants seem to have most overlap with *ELOVL1* associated recessive disorder described in this article. ELOVL4 catalyzes the production of VLCFAs of 28 carbon atoms and more. In concordance with the symptoms seen in patients who carry variants within the *ELOVL4* gene, the highest expression of ELOVL4 is found in brain, retina, and skin.^27^, ^28^ ELOVL1 on the other hand is contributing to the generation of C26‐VLCFAs, which are a substrate of ELOVL4. It, therefore, seems plausible that ELOVL1 deficiency might also cause functional ELOVL4 deficiency because of the lack of C26‐VLCFAs as a substrate for the elongation reaction thereby explaining the overlap of symptoms.

**TABLE 1:** Phenotypes of disorders associated with variants in genes encoding ELOVLs

	ELOVL1 recessive	ELOVL 1 dominant	ELOVL4 recessive (ISQMR)	ELOVL4 dominant (STGD3)	ELOVL4 dominat (SCA34)	ELOVL5 dominant (SCA38)
Congenital onset	+	−	+	−	−	−
Ichthyosis	(+)	+	+	−	(+)	−
Erythrokeratodermia	−	−	−	−	+	−
Spasticity	+	+	+	−	−	−
Seizures	−	−	+	−	−	−
Movement disorder	+	−	−	−	−	−
Ataxia	−	−	−	−	+	+
Dysarthria	+	+	*	−	+	+
ID	(+)	−	+	−		−
Nystagmus	+	+	−	−	+	+
Macular degeneration	−	−	−	+	−	−
Hypomyelination	+	+	+	−	−	−
Thin corpus callosum	+	+	+	−	−	−
Cerebellar atrophy	−	−	−	−	+	+

*Note*: Comparison of ELOVL disorders.

*No language development.

Abbreviations: ISQMR, ichthyosis, spastic quadriplegia, and impaired intellectual development; STGD3, Stargardt‐like macular dystrophy type 3; SCA34, spinocerebellar ataxia 34; SCA38, spinocerebellar ataxia 38; ID, intellectual disability.

## Conclusion

Recessive *ELOVL1* cause a recognizable disorder characterized by ichthyosis, developmental delay, and a movement disorder with progressive spasticity, head tremor, myoclonus, and dystonia. Brain MRI demonstrated that the disorder is part of the group of hypomyelinating leukodystrophies.

## Author Roles

(1) Research Project: A. Conception, B. Organization, C. Execution; (2) Statistical Analysis: A. Design, B. Execution, C. Review and Critique; (3) Manuscript Preparation: A. Writing of the First Draft, B. Review and Critique.

K.M.W.: 1A, 1B, 1C, 2A, 2B, 2C, 3A, 3B.

R.M.: 1A, 1B, 1C, 2C, 3A, 3B.

K.M.: 1B, 1C, 3B.

S.D.: 1B, 1C, 2A, 2B, 3A, 3B.

T.T.: 1B, 1C, 3B.

J.R.: 1B, 1C, 3B.

B.T.: 1B, 1C, 3B.

S.E.: 1C, 2A, 2B, 3B.

A.M.: 1B, 1C, 3B.

C.K.: 1B, 1C, 3B.

N.A.: 1B, 1C, 3B.

W.E.: 1B, 1C, 3B.

A.K.: 1B, 1C, 3B.

M.A.: 1B, 1C, 3B.

JG.: 1A, 1B, 1C, 3B.

B.H.: 1A, 1B, 2A, 2B, 3B.

M.A.: 1B, 1C, 3B.

G.C.: 1B, 1C, 3B.

M.K.: 1B, 1C, 3B.

T.S.: 1B, 1C, 3B.

H.T.: 1C, 2A, 2B, 3B.

P.N.: 1C, 2A, 2B, 3B.

M.H.G.: 1B, 1C, 2A, 2B, 3A, 3B.

H.H.: 1A,1B, 1C, 2C 3A, 3B.

P.H.: 1A, 1B, 1C, 2A, 2B, 2C, 3A, 3B.

## Financial Disclosures

S.D. reports funding from the German Research Foundation (DFG; GA354/14‐1) and the Federal Ministry of Research, Technology and Space (BMFTR; 01GL2402A). K.M. reports funding from the German Research Foundation (DFG; GA354/14‐1, TRR274) and the Federal Ministry of Research, Technology and Space (BMFTR; 01GL2402A)as well as the Research Promotion Program (Forschungsförderungsprogramm) of the University Medical Center Göttingen. J.G. reports grants from the German Research Foundation (DFG; GA354/14‐1, Ga354/16‐1, TRR274, EXC 2067) and grants from the Federal Ministry of Research, Technology and Space (BMFTR; 01GL2402A). J.G. reports personal fees and non‐financial support from Novartis, outside the submitted work. N.A. The Research, Development and Innovation Authority (RDIA), Kingdom of Saudi Arabia (Award # 12996‐iau‐2023‐TAU‐R‐3‐1‐HW‐). R.M., S.E. and H.H. received funding from the Wellcome Trust, the MRC, the MSA Trust, the National Institute for Health Research University College London Hospitals Biomedical Research Centre (NIHR‐BRC), the Michael J Fox Foundation (MJFF), the Fidelity Trust, Rosetrees Trust, the Dolby Family fund, Alzheimer's Research UK (ARUK), MSA Coalition, Parkinson's disease society, Parkinson's Foundation, the Guarantors of Brain, Cerebral Palsy Alliance, FARA, EAN, Victoria Brain bank, the NIH NeuroBioBank, Queen Square BrainBank, the MRC Brainbank Network. P.H. received funding from the German Research foundation (HU 941/12‐5).

## Supporting information


**Video S1.** Supporting Information.

**Video S2.** Supporting Information.

**Video S3.** Supporting Information.

**Video S4.** Supporting Information.

**Figure S1.** Sanger sequencing of the families with ELOVL1 variants. (A) The sequencing analysis of case 1. The red arrow indicates where the mutation occurs. Genomic sequencing reveals that parents and siblings (II.1, II.2, II.3, II4, and II.5) are heterozygous for the c.462G>A variant while one sibling (II.3) doesn't carry the c.462G>A variant. The affected children (patient) has homozygous c.462G>A variant in the ELOVL1 gene. (B) The sequencing analysis of cases 2&3, where both patients show homozygosity for the c.284C>T variant and parents show heterozygosity for the same variant. (C) The sequencing analysis of case 4, where the patient shows homozygosity for the c.457T>C variant, while the parents and one brother show heterozygosity for the c.457T>C variant. Note that all individuals showing heterozygosity for the variants are healthy and Sanger sequencing data for case 5‐7 is unavailable.

**Figure S2.** Multiple sequence alignment of isoform 1 of ELOVL1 protein across several species. Alignment illustrates similarities and differences in ELOVL1, indicating evolutionary conservation and species‐specific variations. Conserved regions are marked with asterisks (*), while specific mutations are highlighted in bold. Sequence alignment was performed using the ClustalX.

**Table S1.** Clinical and genetic findings of 7 patients reported in this study and 4 cases reported previously.

**Table S2.** LCFAs and VLCFAs (nmol/mL) concentration in patient 1 (c.462G>A) and the healthy sibling's plasma.

**Table S3.** Annotations and predictions of *ELOVL1* variants reported in this study.

**Table S4.** Primer sequences used in this study.

**Table S5.** Chromatographic conditions.

## Data Availability

The tabulated data that support the findings of this study are available from the corresponding 13 authors, on reasonable request from a qualified investigator.
