# Supplementary material for: Biallelic ELOVL1 Variants Are Linked to Hypomyelinating Leukodystrophy, Movement Disorder, and Ichthyosis
Source: Mov Disord. 2025 Jul 1;40(9):1836–50. doi: 10.1002/mds.30258 (PMC12485584; doi:10.1002/mds.30258)
Supplement: Supplementary file 5 — Figure S1. Sanger sequencing of the families with ELOVL1 variants. (A) The sequencing analysis of case 1. The red arrow indicates where the mutation occurs. Genomic sequencing reveals that parents and siblings (II.1, II.2, II.3, II4, and II.5) are heterozygous for the c.462G>A variant while one sibling (II.3) doesn't carry the c.462G>A variant. The affected children (patient) has homozygous c.462G>A variant in the ELOVL1 gene. (B) The sequencing analysis of cases 2&3, where both patients show homozygosity for the c.284C>T variant and parents show heterozygosity for the same variant. (C) The sequencing analysis of case 4, where the patient shows homozygosity for the c.457T>C variant, while the parents and one brother show heterozygosity for the c.457T>C variant. Note that all individuals showing heterozygosity for the variants are healthy and Sanger sequencing data for case 5‐7 is unavailable. [file MDS-40-1836-s011.pdf]

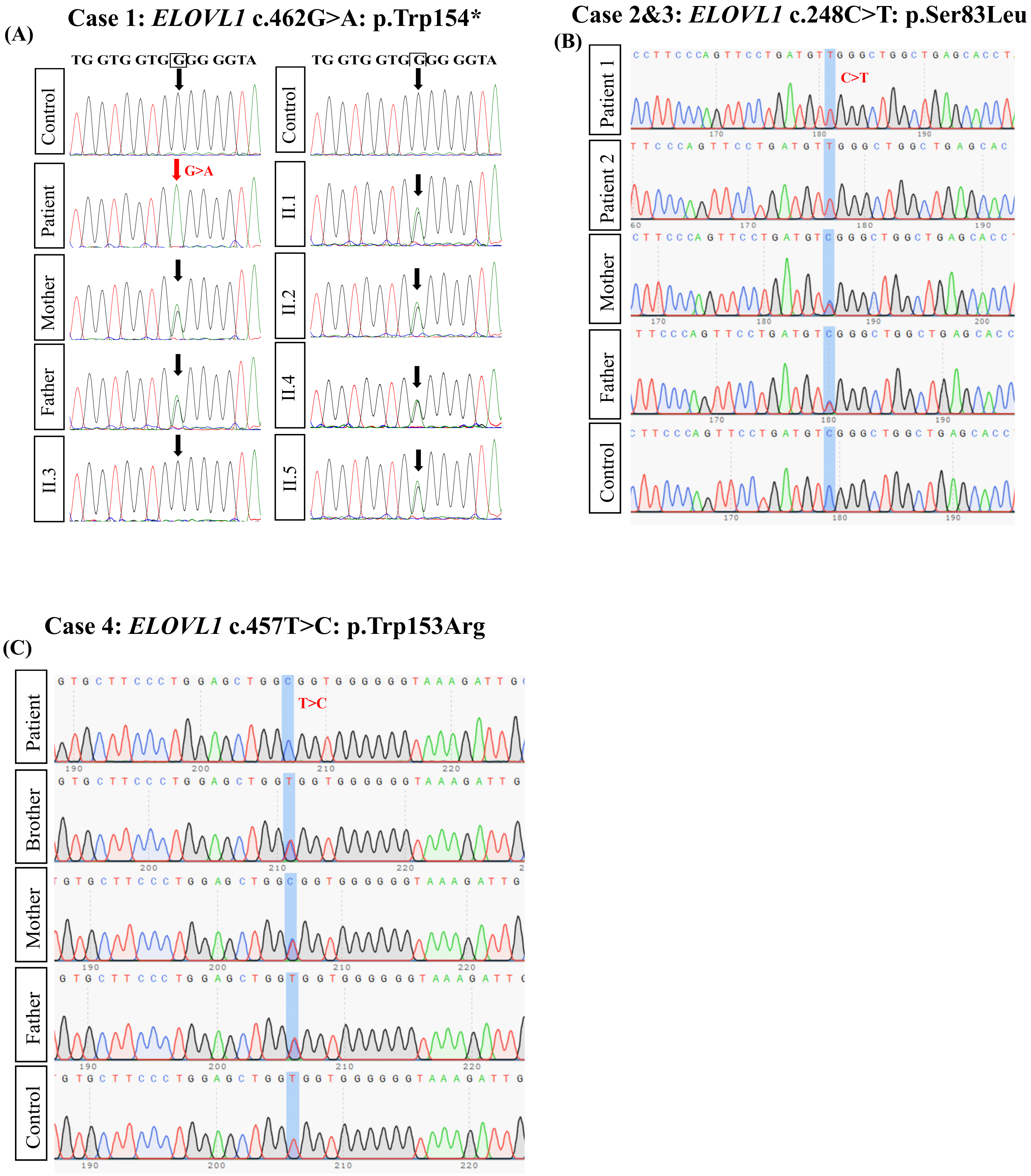

Supplemental figure S1. Sanger sequencing of the families with ELOVL1 variants. (A) The sequencing analysis of case 1. The red arrow indicates where the mutation occurs. Genomic sequencing reveals that parents and siblings (II.1, II.2, II.3, II4, and II.5) are heterozygous for the c.462G>A variant while one sibling (II.3) doesn't carry the c.462G>A variant. The affected children (patient) has homozygous c.462G>A variant in the *ELOVL1* gene. (B) The sequencing analysis of cases 2&3, where both patients show homozygosity for the c.284C>T variant and parents show heterozygosity for the same variant. (C) The sequencing analysis of case 4, where the patient shows homozygosity for the c.457T>C variant, while the parents and one brother show heterozygosity for the c.457T>C variant. Note that all individuals showing heterozygosity for the variants are healthy and Sanger sequencing data for case 5 is unavailable
