# Supplementary material for: Biallelic ELOVL1 Variants Are Linked to Hypomyelinating Leukodystrophy, Movement Disorder, and Ichthyosis
Source: Mov Disord. 2025 Jul 1;40(9):1836–50. doi: 10.1002/mds.30258 (PMC12485584; doi:10.1002/mds.30258)
Supplement: Supplementary file 6 — Figure S2. Multiple sequence alignment of isoform 1 of ELOVL1 protein across several species. Alignment illustrates similarities and differences in ELOVL1, indicating evolutionary conservation and species‐specific variations. Conserved regions are marked with asterisks (*), while specific mutations are highlighted in bold. Sequence alignment was performed using the ClustalX. [file MDS-40-1836-s009.docx]

**R18W** **S83L**

NP_073732.1 **[Homo sapiens (human)]** MEAVVNLYQEVMKHADPRIQGYPLMGSPLLMTSILLTYVYFVLSLGPRIM 50 ANRKPFQLRGFMIVYNFSLVALSLYIVYEFLM**S**GWLSTYTWRCDPVDYSN 100

NP_001411917.1 **[Mus musculus (house mouse)]** MEAVVNLYHELMKHADPRIQSYPLMGSPLLITSILLTYVYFILSLGPRIM 50 ANRKPFQLRGFMIVYNFSLVILSLYIVYEFLM**S**GWLSTYTWRCDPIDFSN 100

NP_001037740.1 **[Rattus norvegicus (rat)]**  MEAVVNLYQELMKCADPRIQNYPLMGSPLLITSILLTYVYFVLSLGPRIM 50 ANRKPFQLRGFMIVYNFSLVTLSLYIVYEFLM**S**GWLSTYTWRCDPVDFSN 100

NP_001248488.1 **[Macaca mulatta (rhesus monkey)]** MEAVMNLYQEMMKHADPRIQGYPLMGSPLLMTSILLTYVYFVLSLGPRIM 50 ANRKPFQLRGFMIVYNFSLVAFSLYIVYEFLM**S**GWLSTYTWRCDPVDYSN 100

XP_038543917.1**_[Canis lupus familiaris (dog)]** MEAVVNLYQEMMKYADPRLQGYPLMGSPLLMTSILLTYVYFVLSLGPRIM 50 ANRKPFQLRGFMIVYNFSLVALSLYIVYEFLM**S**GWLSTYTWRCDPVDYSN 100

XP_006934720.1**_[Felis catus (domestic cat)]** MEAVVNLYQEMMKYADPRVQGYPLMGSPLLMTSILLTYVYFVLSLGPRIM 50 ANRKPFQLRGFMIVYNFSLVALSLYIVYEFLM**S**GWLSTYTWRCDPVDYSN 100

XP_014592857.1**_[Equus caballus (horse)]** MEAVVNLYQEMMKCADPRIQGYPLMGSPLLMTSILLTYVYFVLSLGPRIM 50 ANRKPFQLRSFMIVYNFSLVALSLYIVYEFLM**S**GWLSTYTWRCDPVDYSN 100

****:***:*:** ****:*.*********:**********:******** *********.********** :***********************:*:**

**W154* S165F**

**W153R G164D**

NP_073732.1 **[Homo sapiens (human)]**  SPEALRMVRVAWLFLFSKFIELMDTVIFILRKKDGQVTFLHVFHHSVLPW 150 SW**WW**GVKIAPGGM**GS**FHAMINSSVHVIMYLYYGLSAFGPVAQPYLWWKKH 200

NP_001411917.1 **[Mus musculus (house mouse)]** SPEALRMVRVAWLFMLSKVIELMDTVIFILRKKDGQVTFLHVFHHSVLPW 150 SW**WW**GIKIAPGGM**GS**FHAMINSSVHVVMYLYYGLSALGPVAQPYLWWKKH 200

NP_001037740.1 **[Rattus norvegicus (rat)]** NPEALRMVRVAWLFMLSKVIELMDTVIFILRKKDGQVTFLHVFHHSVLPW 150 SW**WW**GIKIAPGGM**GS**FHAMINSSVHVVMYLYYGLSALGPVAQPYLWWKKH 200

NP_001248488.1 **[Macaca mulatta (rhesus monkey)]** SPEALRMVRVAWLFLFSKFIELMDTVIFILRKKDGQVTFLHVFHHSVLPW 150 SW**WW**GVKIAPGGM**GS**FHAMINSSVHVIMYLYYGLSAIGPVAQPYLWWKKH 200

XP_038543917.1**_[Canis lupus familiaris (dog)]** SPEALRMVRVAWLFLFSKFIELMDTVIFILRKKDGQVTFLHVFHHSVLPW 150 SW**WW**GVKIAPGGM**GS**FHAMINSSVHVVMYLYYGLSALGPVAQPYLWWKKH 200

XP_006934720.1**_[Felis catus (domestic cat)]** SPEALRMVRVAWLFLFSKFIELMDTVIFILRKKDGQVTFLHVFHHSVLPW 150 SW**WW**GVKIAPGGM**GS**FHAMINSSVHVVMYLYYGLSALGPVAQPYLWWKKH 200

XP_014592857.1**_[Equus caballus (horse)]** NPEALRMVRVAWLFLFSKFIELMDTVIFILRKKDGQVTFLHVFHHSVLPW 150 SW**WW**GVKIAPGGM**GS**FHAMINSSVHVVMYLYYGLSAIGPAAQPYLWWKKH 200

.*************::**.******************************* *****:********************:*********:**.**********

NP_073732.1 **[Homo sapiens (human)]** MTAIQLIQFVLVSLHISQYYFMSSCNYQYPVIIHLIWMYGTIFFMLFSNF 250 WYHSYTKGKRLPRALQQNGAPGIAKVKAN 279

NP_001411917.1 **[Mus musculus (house mouse)]** MTAIQLIQFVLVSLHISQYYFMPSCNYQYPIIIHLIWMYGTIFFILFSNF 250 WYHSYTKGKRLPRAVQQNGAPATTKVKAN 279

NP_001037740.1 **[Rattus norvegicus (rat)]** MTAIQLIQFVLVSLHISQYYFMPSCNYQYPIIIHLIWMYGTIFFILFSNF 250 WYHSYTKGKRLPRAVQQNGAAASMKVKAN 279

NP_001248488.1 **[Macaca mulatta (rhesus monkey)]** MTAIQLIQFVLVSLHISQYYFMSSCNYQHPVIIHLIWMYGTIFFMLFSNF 250 WYHSYTKGKRLPRALQQNGAPGIAKVKAN 279

XP_038543917.1**_[Canis lupus familiaris (dog)]** MTAIQLIQFVLVSLHISQYYFMPSCDYQYPVIIHLIWMYGTIFFVLFSNF 250 WYHSYTKGKRLPRVLQ-NGAPGTAKVKAN 278

XP_006934720.1**_[Felis catus (domestic cat)]**  MTAIQLIQFVLVSLHISQYYFMPSCNYQYPVIIHLIWMYGTIFFVLFSNF 250 WYHSYTKGKRLPRVLQQNGAPGTAKVKAN 279

XP_014592857.1**_[Equus caballus (horse)]** MTAIQLIQFVLVSLHISQYYFMPSCNYQYPVIIHLIWMYGTIFFGLFSNF 250 WYQSYTKGKRLPRVLQQNGVPGTAKVKAN 279

**********************.**:**:*:************* ***** **:**********.:* **... *****

Supplementary Figure 2: Multiple sequence alignment of isoform 1 of ELOVL1 protein across several species. Alignment illustrates similarities and differences in ELOVL1, indicating evolutionary conservation and species-specific variations. Conserved regions are marked with asterisks (*), while specific mutations are highlighted in bold. Sequence alignment was performed using the ClustalX.
