# Supplementary material for: Biallelic ELOVL1 Variants Are Linked to Hypomyelinating Leukodystrophy, Movement Disorder, and Ichthyosis
Source: Mov Disord. 2025 Jul 1;40(9):1836–50. doi: 10.1002/mds.30258 (PMC12485584; doi:10.1002/mds.30258)
Supplement: Supplementary file 7 — Table S1. Clinical and genetic findings of 7 patients reported in this study and 4 cases reported previously. [file MDS-40-1836-s008.docx]

**Supplementary table 1: Clinical and genetic findings of 7 patients reported in this study and 4 cases reported previously**

|  | case 1 | case 2 | case 3 | case 4 | case 5 | Case 6 | Case 7 | T. Takahashi et al. 2022 | T. Takahashi et al. 2022 | Mueller et al. 2019, Kutkowska-Kazmierczak et al 2019 | Mueller et al. 2019, Kutkowska-Kazmierczak et al 2019 |
| --- | --- | --- | --- | --- | --- | --- | --- | --- | --- | --- | --- |
| DNA | c.462G>A, homozygous | c.248C>T homozygous | c.248C>T homozygous | c.457T>C homozygous | c.491G>A  homozygous | c.52C>T  homozygous | c.52C>T  homozygous | c.376-2A>G homozygous | c.376-2A>G homozygous | c.494C>T heterozygous | c.494C>T heterozygous |
| Protein | p.Trp154* | p.Ser83Leu | p.Ser83Leu | p.Trp153Arg | p.Gly164Asp | p.Arg18Trp | p.Arg18Trp | Skipping of exon 6 | Skipping of exon 6 | p.Ser165Phe | p.Ser165Phe |
| Consanguinity | yes | yes | yes | yes | yes | yes | yes | yes | yes | no | no |
| Age (y) | 14 | 7 | 4 | 6 | 13 | 21 | 20 | 9 | 5 | 15 | 5 |
| Gender | F | M | M | F | F | M | F | F | M | M | M |
| Congenital ichthyosis | + | - | - | + | + | + | + | + | + | - | - |
| Ichthyosis trunk | + | - | - | + | + | + | + | + | + | + | + |
| Ichthyosis limb | + | - | - | + | + | + | + | + | + | + | + |
| Motor DD | + | + | + | + | + | + | + | + | + | + | + |
| Sitting | + | + | + | + | + | + | + | + | + | + | + |
| Walking | + | + | - | - | + | - | - | - | - | - | (+) |
| Hyperreflexia | + | + | + | + | + | + | + | + | + | + | + |
| Axial hypotonia | + | + | + | + | + | NN | + | NN | NN | - | - |
| Spasticity limbs | + | + | + | + | + | + | + | + | + | + | + |
| Joint contractures | - | + | + | - | + | + | + | + | + | + | + |
| Movement disorder | + | + | + | + | + | + | + | + | + | - | - |
| Head tremor | + | + | + | + | + | + | + | + | + | - | - |
| Dystonia | + | + | + | - | + | - | - | NN | NN | - | - |
| Myoclonus | + | + | + | + | - | + | + | + | + | - | - |
| Dysarthria | + | + | + | NN | + | + | + | + | + | + | + |
| Photophobia | + | - | - | NN | + | - | - | NN | NN | + | + |
| Nystagmus | - | + | + | NN | + | + | + | + | + | + | + |
| hearing impairment | - | - | - | NN | - | NN | NN | + | NN | + | + |
| ID | + | + | + | - | + | NN | NN | - | - | - | - |
| Loss of motor abilities | + | + | + | + | + | + | + | + | NN | + | + |
| Cerebral hypomyelination | + | + | + | + | + | NN | + | + | + | + | + |
| Cerebellar hypomyelination | + | + | + | + | + | NN | - | + | + | - | - |
| Hypoplastic corpus callosum | + | + | + | + | + | NN | - | NN | NN | + | + |

ID: intellectual disability; DD: Developmental delay; NN not known; y: years
