# Supplementary material for: Biallelic ELOVL1 Variants Are Linked to Hypomyelinating Leukodystrophy, Movement Disorder, and Ichthyosis
Source: Mov Disord. 2025 Jul 1;40(9):1836–50. doi: 10.1002/mds.30258 (PMC12485584; doi:10.1002/mds.30258)
Supplement: Supplementary file 8 — Table S2. LCFAs and VLCFAs (nmol/mL) concentration in patient 1 (c.462G>A) and the healthy sibling's plasma. [file MDS-40-1836-s007.docx]

**Supplementary Table 2:** LCFAs and VLCFAs (nmol/mL) concentration in patient 1 (c.462G>A) and the healthy sibling’s plasma

|  | Patient | Sibling 1 | Sibling 2 | Sibling 3 | Sibling 4 |
| --- | --- | --- | --- | --- | --- |
| C16:0 | 9.2 | 8 | 8.3 | 7.6 | 7.1 |
| C18:0 | 16.4 | 15.4 | 17.1 | 15.5 | 18.7 |
| C22:0 | 41.4 | 32.5 | 47.4 | 30.9 | 0.41 |
| C24:0 | 18 | 26.5 | 30.9 | 32.7 | 29.5 |
| C26:0 | 0.21 | 0.34 | 0.41 | 0.68 | 0.44 |
| C24:0/ C22:0 ratio | 0.43 | 0.82 | 0.65 | 0.64 | 0.65 |
| C26:0/ C22:0 ratio | 0.005 | 0.010 | 0.009 | 0.013 | 0.010 |
| Phytanic acid | 3.1 | 4.9 | 3.8 | 3.1 | 10.2 |
| Pristanic acid | <1 | <1 | <1 | <1 | 1.7 |
