# Supplementary material for: Biallelic ELOVL1 Variants Are Linked to Hypomyelinating Leukodystrophy, Movement Disorder, and Ichthyosis
Source: Mov Disord. 2025 Jul 1;40(9):1836–50. doi: 10.1002/mds.30258 (PMC12485584; doi:10.1002/mds.30258)
Supplement: Supplementary file 9 — Table S3. Annotations and predictions of ELOVL1 variants reported in this study. [file MDS-40-1836-s004.docx]

Supplementary table 3: Annotations and predictions of *ELOVL1* variants reported in this study

| **Variant Annotation** | **case 1** | **case 2 + 3** | **case 4** | **case 5** | **case 6 + 7** | **T. Takahashi et al. 2022** | **Mueller et al. 2019, Kutkowska-Kazmierczak et al 2019** |  |
| --- | --- | --- | --- | --- | --- | --- | --- | --- |
| Genomic Position  chr 1 (hg38) | 43364561C>T | 43364998G>A | 43364566A>G | 43364451C>T | 43365371G>A | 43364649T>C | 43364448G>A |  |
| cDNA Position  (NM_022821.3) | c.462G>A | c.248C>T | c.457T>C | c.491G>A | c.52C>T | c.376-2A>G | c.494C>T |  |
| Protein Change  (NP_073732.1) | p.Trp154* | p.Ser83Leu | p. Trp153Arg | p.Gly164Asp | p.Arg18Trp | - | p.Ser165Phe |  |
| Exon | 6 | 4 | 6 | 7 | 3 | - | 7 |  |
| Consequence | Nonsense | Missense | Missense | Missense | Missense | Splice site | Missense |  |
| Zygosity | Homozygous | Homozygous | Homozygous | Homozygous | Homozygous | Homozygous | Homozygous |  |
| **Variant frequency** | | | | | | | | |
| gnomAD v4.1.0  AF [ Het; Hom] | - | 0.000001240  [2 of 1613262; 0] | - | - | 0.000003724  [6 of 1610972; 0] | - | - |  |
| **Conservation and Functional Impact Scores** | | | | | | | | |
| GERP (-12.3 – 6.17) | 5.66 | 5.77 | 5.66 | 5.56 | 5.95 | 5.88 | 5.56 |  |
| CADD | 38 | 27.2 | 32 | 24.0 | 31 | 35 | 24.7 |  |
| Polyphen-2 (0 – 1) | NA | Probably damaging (HumDiv: 0.966) | Probably damaging (HumDiv: 1.0) | Probably damaging (HumDiv: 1.0) | Probably damaging (HumDiv: 1.0) | NA | Benign  (HumDiv: 0.425) |  |
| SIFT (0 – 1) | NA | Damaging (0.005) | Damaging (0.000) | Damaging (0.001) | Damaging (0.000) | NA | Damaging (0.000) |  |
| Provean (-14 – 14) | NA | Damaging (-4.99) | Damaging (-13.67) | Damaging (-4.73) | Damaging (-7.47) | NA | Damaging (-3.28) |  |
| MutationTaster (0 – 1) | Disease Causing  (1) | Disease Causing  (1) | Disease Causing  (1) | Disease Causing  (1) | Disease Causing  (1) | Disease Causing (1) | Disease Causing  (1) |  |
| AlphaMissense  (0 – 1) | NA | Likely pathogenic (0.783) | Likely pathogenic (0.997) | Likely pathogenic (0.983) | Likely pathogenic (0.980) | NA | Likely pathogenic (0.985) |  |
| ADA (0 – 1) | NA | NA | NA | NA | NA | 0.999 | NA |  |
| RF (0 – 1) | NA | NA | NA | NA | NA | 0.808 | NA |  |
