# Supplementary material for: Biallelic ELOVL1 Variants Are Linked to Hypomyelinating Leukodystrophy, Movement Disorder, and Ichthyosis
Source: Mov Disord. 2025 Jul 1;40(9):1836–50. doi: 10.1002/mds.30258 (PMC12485584; doi:10.1002/mds.30258)
Supplement: Supplementary file 10 — Table S4. Primer sequences used in this study. [file MDS-40-1836-s010.docx]

(A) Primer for Sanger Sequencing validation

| Gene | Forward primer 5’-3’ | Reverse primer 5’-3’ | Target |
| --- | --- | --- | --- |
| *ELOVL1* | CCACATCCCTTCCCAGTTCC | GAGGGGAAGAGGGGGTAGAG | gDNA |

(B) Primer for RT-qPCR

| Gene | Forward primer 5’-3’ | Reverse primer 5’-3’ |
| --- | --- | --- |
| *ELOVL1* | TCTCACTTGGGCCTCGCATC | CAGCCCGACATCAGGAACTCATA |
| *ELOVL2* | ATGTTTGGACCGCGAGATT | TGTATCTGCTCTCAATATGGCTG |
| *ELOVL3* | GCAAACCGTGTGCTTCATC | GCTGTGTCTCCGAGTTCTATG |
| *ELOVL4* | CCTGGTCCATCGCAGATAAG | AGCCACACAAACAGGAGATAA |
| *ELOVL5* | GGCCCTCGAGATACTAGAGTAAA | TTGGTCCCAGCCATACAATTAG |
| *ELOVL6* | CAATGGACCTGTCAGCAAATTC | GATACCAGTGCAGGAAGATCAG |
| *GAPDH* | CAGCCGCATCTTCTTTTGCG | GCCCAATACGACCAAATCCGT |

**Supplementary table 4: Primer sequences used in this study**
