# Supplementary material for: Biallelic ELOVL1 Variants Are Linked to Hypomyelinating Leukodystrophy, Movement Disorder, and Ichthyosis
Source: Mov Disord. 2025 Jul 1;40(9):1836–50. doi: 10.1002/mds.30258 (PMC12485584; doi:10.1002/mds.30258)
Supplement: Supplementary file 11 — Table S5. Chromatographic conditions. [file MDS-40-1836-s003.docx]

**Supplementary Table 5: Chromatographic conditions**

| Time (min) | Flow (µL) | Methanol (%) | 1 % Formic acid in H_2_O |
| --- | --- | --- | --- |
| 0.0 | 400 | 10 | 90 |
| 0.01 | 400 | 100 | 0 |
| 3.0 | 400 | 100 | 0 |
| 5.0 | 800 | 100 | 0 |
| 7.0 | 800 | 100 | 0 |
| 7.01 | 800 | 10 | 90 |
| 7.8 | 800 | 10 | 90 |
| 8.3 | 300 | 10 | 90 |
| 9.5 | 300 | 10 | 90 |
| 9.51 | 400 | 10 | 90 |
| 10.5 | 400 | 10 | 90 |
